# Endoscopic intermuscular dissection for a duodenal neuroendocrine tumor using saline-immersion therapeutic endoscopy

**DOI:** 10.1055/a-2621-2885

**Published:** 2025-07-04

**Authors:** Priscilla Lopez, Mohan Ramchandani, Sundeep Lakhtakia, Pradev Inavolu, Hardik Rughwani, Anjan Kaipa, D. Nageshwar Reddy

**Affiliations:** 1 78470 Department of Gastroenterology, Asian Institute of Gastroenterology, Hyderabad, India

A 68-year-old man with type 2 diabetes and hypertension underwent upper gastrointestinal endoscopy for anemia, revealing an ulcerated subepithelial lesion on the anterior-superior wall of the duodenal bulb ( Fig. 1 **a** ). Endoscopic ultrasound revealed a 25-mm hypoechoic lesion arising from the submucosa and extending into the muscularis propria, with increased vascularity ( Fig. 1 **b, c** ).

**Fig. 1:**
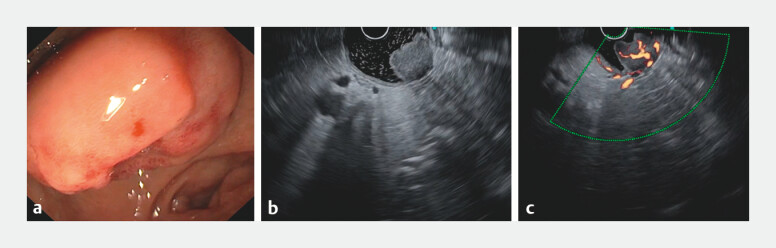
Initial investigations. **a** Ulcerated subepithelial lesion in the duodenal bulb (D1). **b** Endoscopic ultrasound showing a 25-mm hypoechoic lesion arising from the submucosa and extending into the muscularis propria. **c** Endoscopic ultrasound with Doppler demonstrating increased vascularity within the lesion.

Computed tomography (CT) and positron emission tomography-CT confirmed a localized lesion without nodal or distant spread ( Fig. 2 ). Based on these features, endoscopic full-thickness resection (EFTR) was planned.

**Fig. 2:**
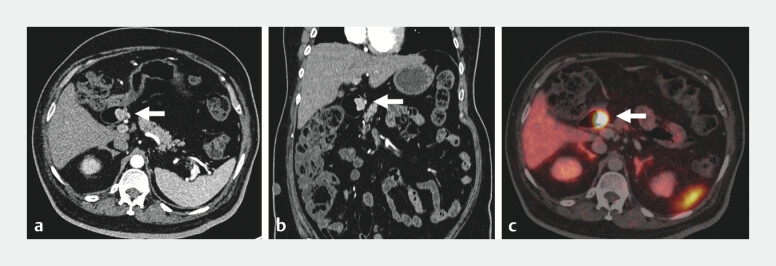
Computed tomography (CT) imaging. **a–c** Ga-68 DOTA-TOC positron emission tomography with CT showing a tracer-avid (maximum standardized uptake value: 178.3), well-defined, intensely arterially enhancing polypoidal soft tissue lesion (arrow) in the duodenum (D1) along the medial aspect.

The procedure was performed under general anesthesia. Initial dissection with conventional EFTR using carbon dioxide insufflation was limited by poor maneuverability, presence of fibrosis, and bleeding. The approach was converted to saline-immersion therapeutic endoscopy (SITE). Swift Coagulation (effect 3.5) was used to safely coagulate vessels without a coagulation grasper, minimizing the risk of perforation.

An intermuscular dissection technique was employed to target the space between the inner and outer muscularis propria, avoiding EFTR when possible. Only a <6-mm area required EFTR due to deep invasion. This approach minimized peritoneal exposure and avoided pneumoperitoneum ( Fig. 3 , Video 1 ). The resection site was closed using the loop-and-clip technique. No complications occurred.

**Fig. 3:**
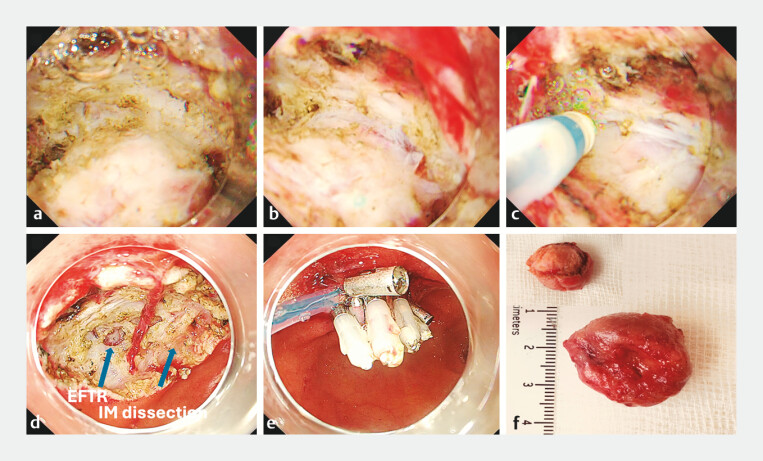
Endoscopy images. **a–c** Endoscopic intermuscular dissection using saline-immersion therapeutic endoscopy, showing the space between the inner and outer muscularis propria. **d** Resection site demonstrating predominant intermuscular dissection with a small area (<6 mm) of endoscopic full-thickness resection where the lesion extended into the muscularis propria (arrows). **e** Defect closed using the loop-and-clip technique. **f** Resected tumor.

Saline immersion and intermuscular dissection enabled safe resection of a duodenal neuroendocrine tumor involving the muscularis propria, minimizing full-thickness resection and preventing procedural complications.Video 1

Histology confirmed a well-differentiated neuroendocrine tumor, infiltrating the muscularis propria, with negative lateral and vertical margins and no lymphovascular invasion ( Fig. 4 ).

**Fig. 4:**
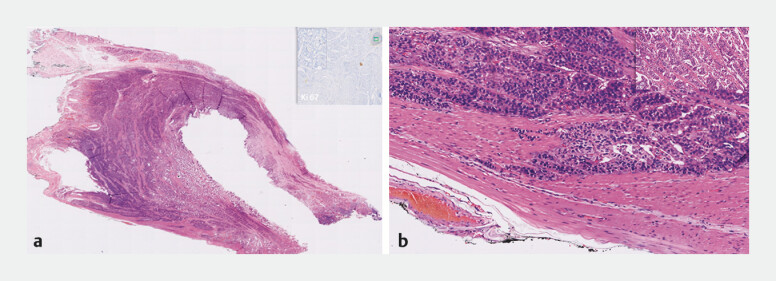
Histology. **a** Well-differentiated (G1) neuroendocrine tumor with Ki-67 index <2%. **b** Negative resection margins; inset demonstrates tumor cell infiltration into the muscularis propria.

Endoscopic resection of duodenal lesions involving the muscularis propria is challenging due to the thin wall, narrow lumen, and proximity to important vessels 1 . While EFTR offers an alternative to surgery, it carries risks such as pneumoperitoneum and bleeding 2 .

This video demonstrates a combined approach using the advantages of SITE-enhanced visualization, elimination of gas insufflation, buoyancy-assisted traction, and reduced thermal injury through gradual coagulation enabled by improved conductivity 3 4 (“frozen tree” effect). Furthermore, intermuscular dissection 5 enabled a targeted approach, limiting EFTR to only the extent necessary for complete tumor removal, highlighting its value in anatomically challenging cases.

Endoscopy_UCTN_Code_TTT_1AO_2AC

## Notes

Endoscopy E-Videos https://eref.thieme.de/e-videos
*E-Videos* is an open access online section of the journal *Endoscopy* , reporting on interesting cases and new techniques in gastroenterological endoscopy. All papers include a high-quality video and are published with a Creative Commons CC-BY license. Endoscopy E-Videos qualify for HINARI discounts and waivers and eligibility is automatically checked during the submission process. We grant 100% waivers to articles whose corresponding authors are based in Group A countries and 50% waivers to those who are based in Group B countries as classified by Research4Life (see: https://www.research4life.org/access/eligibility/ ). This section has its own submission website at https://mc.manuscriptcentral.com/e-videos .
